# Lower socioeconomic status is associated with increased long‐term mortality after sudden cardiac arrest

**DOI:** 10.1002/clc.23211

**Published:** 2019-06-04

**Authors:** Ankit N. Medhekar, Shubash Adhikari, Ahmed S. Abdul‐Al, Sayna Matinrazm, Krishna Kancharla, Aditya Bhonsale, Sandeep K. Jain, Samir Saba

**Affiliations:** ^1^ Department of Medicine University of Pittsburgh Medical Center Pittsburgh Pennsylvania; ^2^ Heart and Vascular Institute University of Pittsburgh Medical Center Pittsburgh Pennsylvania

**Keywords:** household income, mortality, socioeconomic status, sudden cardiac arrest

## Abstract

**Background:**

Determinants of long‐term survival after sudden cardiac arrest (SCA) are not fully elucidated. We investigated the impact of patients' socioeconomic status (SES) on long‐term mortality in SCA survivors.

**Objective:**

To investigate the association between SES, as estimated by median household income by zip code of residence, and long‐term survival after SCA.

**Methods:**

We analyzed the electronic medical records of patients who presented to our institution with SCA between 2000 and 2012 and were discharged alive. Patients were stratified into quartiles by median household income of their home zip code. Baseline characteristics of patients were compared by income quartiles. The impact of SES on mortality was assessed using a multivariable Cox proportional hazards model incorporating all unbalanced covariates.

**Results:**

Our cohort consisted of 1420 patients (mean age of 62 years; 41% men; 82% white). Over a 3.6‐year median follow‐up, 47% of patients died. After adjusting for unbalanced baseline covariates, patients in the poorest income quartile had a 25% increase in their risk of death compared to other SCA survivors (hazard ratios = 1.25, 95% confidence interval 1.00‐1.56, *P* = .046).

**Conclusion:**

In conclusion, lower SES is an independent predictor of long‐term mortality in survivors of SCA. Designing interventions to improve survival after SCA requires attention to patients' social and economic factors.

## 1 INTRODUCTION

Sudden cardiac arrest (SCA) is an important cause of morbidity and mortality in the United States; however, the impact of socioeconomic status (SES) on survival after SCA remains incompletely elucidated. Each year, about 300 000 Americans experience an out‐of‐hospital SCA.^1^ Despite drastic improvements in care for patients with SCA, 1‐year survival can be as low as 14%.^2^ Identifying patients who are at increased risk for mortality after SCA can help target future interventions and improve outcomes.

SES is an important determinant of long‐term outcomes in cardiovascular disease.^3^, ^4^, ^5^, ^6^, ^7^ The effect of SES on outcomes has been found to be independent of the indicator used to assess SES.^8^ This effect has also been documented in countries with publicly funded healthcare systems,^6^, ^9^, ^10^, ^11^ suggesting mechanisms that transcend patients' health care coverage. A handful of prior studies have been performed that suggest that lower SES is associated with higher incidence of SCA.^12^, ^13^, ^14^ However, little work has been performed to examine the influence of SES on outcomes after SCA.

In the present study, we sought to determine the effect of lower SES, as estimated by median household income of the patient's zip code of residence, on long‐term mortality in a cohort of patients that initially presented to our institution with SCA and were discharged alive. We hypothesized that, compared to patients with higher SES, those with lower SES would have higher mortality.

## 2 METHODS

We performed a retrospective review of SCA survivors who were admitted to hospitals of the University of Pittsburgh Medical Center between 2000 and 2012. Patients with International Classification of Disease, ninth Revision (ICD‐9) codes for ventricular fibrillation (427.41), ventricular flutter (427.42), ventricular tachycardia (427.1), and cardiac arrest (427.5) who were 18 years of age or older at the time of the SCA and who did not have an implantable cardioverter‐defibrillator (ICD) were identified. The electronic records of these patients were then manually reviewed to confirm the presence of SCA and the absence of prior ICD. Demographic and clinical data for these patients were abstracted from the electronic health records.

SES is multifactorial, incorporating elements of patients' income, employment status, career, and education level among other factors. For the purpose of the present analysis, we used the median household income of the patient's zip code of residence as a proxy for SES as commonly performed in the literature.^12^, ^15^, ^16^ To determine this value, we used data from 2012 to 2016 American Community Survey 5‐year estimates for median household income. The American Community Survey is an ongoing, yearly survey collected by the United States Census Bureau. Data are collected by mail, but follow‐ups included telephone calls and personal visits, except during 2013, in which October housing units did not have a telephone or personal follow‐up because of the government shut down.^17^

All‐cause mortality was the primary outcome of this study. We assessed it by examining the rate of mortality during follow‐up as well as the time to death. Mortality was ascertained by querying the electronic medical records and the social security death index using the updated Social Security Administration Death Master file, for which our healthcare system is exempt from the 3‐year delay period by the Social Security Administration.

A univariate analysis using the Pearson χ^2^ test, Student *t* test, and Mann‐Whitney *U* test was performed as appropriate to determine baseline differences for clinical and demographic variables between income quartiles. Variables that were significantly different among the income quartiles at the *P* < .10 level in the univariate analysis were included in subsequent analyses. A multivariable logistic regression model was generated to determine the odds ratio of mortality after SCA for patients in the lowest income quartile compared to other SES quartiles. Similarly, the time to death was compared between patients in the lowest SES quartile and those in the highest three quartiles using Kaplan‐Meier analysis and the log‐rank test. A Cox regression model was used to adjust for unbalanced covariates. Two‐sided *P*‐values .05 were considered statistically significant. The majority of statistical analyses were performed using the statistical software package SPSS, version 25.0 (IBM, Armonk, New York).

## 3 RESULTS

We identified 1420 patients who were discharged alive after SCA from 2000 to 2012. These patients were followed for a mean duration of 3.8 ± 3.1 years (median 3.6 years). Of the patients identified, approximately 41% were male, 82% were white, and the average age was 62 years. Patients from the lowest quartile were more likely to be black, have pulmonary disease, and have a history of tobacco and alcohol use. Complete baseline characteristics by income quartile are detailed in Table 1.

**Table 1:** Baseline characteristics by quartiles of household income

Characteristic	Quartile 1	Quartile 2	Quartile 3	Quartile 4	*P*‐value
Age at SCA (mean ± SD) years	60.7 ± 16.0	63.7 ± 14.5	61.8 ± 16.4	62.5 ± 15.5	.016
Sex—no. (%)					.75
Sex—women, no. (%)	209 (57.3)	210 (58.5)	203 (59.5)	217 (61.1)	.75
Race/ethnicity—no. (%)					<.001
White	238 (65.2)	308 (85.8)	294 (86.2)	330 (93.0)	
Black	105 (28.8)	25 (7.0)	25 (7.3)	15 (4.2)	
Other	22 (6.0)	26 (7.2)	22 (6.5)	10 (2.8)	
BMI (mean ± SD)	29.1 ± 7.4	29.7 ± 8.5	30.1 ± 7.1	30.4 ± 8.3	.20
Blood pressure (mean ± SD) mm Hg					
Systolic	129.9 ± 33.9	126.3 ± 31.1	124.3 ± 30.5	127.0 ± 29.4	.12
Diastolic	72.8 ± 22.8	68.8 ± 20.6	69.1 ± 21.1	72.0 ± 36.3	.10
History—no. (%)					
Atrial fibrillation	96 (26.3)	120 (33.4)	109 (32.0)	108 (30.4)	.31
Myocardial infarction/ischemia	125 (34.2)	152 (42.3)	128 (37.5)	135 (38.0)	.56
Diabetes mellitus	121 (33.2)	125 (34.8)	107 (31.4)	108 (30.4)	.30
Pulmonary disease	143 (39.2)	118 (32.9)	94 (27.6)	107 (30.1)	.003
Chronic kidney disease	57 (15.6)	65 (18.1)	58 (17.0)	50 (14.1)	.52
Hypertension	242 (66.3)	212 (59.1)	210 (61.6)	212 (59.7)	.13
Previous coronary artery disease	224 (61.4)	241 (67.1)	203 (59.5)	238 (67.0)	.39
Cerebrovascular disease	36 (9.9)	37 (10.3)	40 (11.7)	53 (14.9)	.029
Peripheral vascular disease	30 (8.2)	48 (13.4)	29 (8.5)	41 (11.5)	.46
Malignancy	40 (11.0)	40 (11.1)	41 (12.0)	36 (10.1)	.830
Tobacco	149 (40.8)	128 (35.7)	104 (30.5)	98 (27.6)	<.001
Alcohol	77 (21.1)	58 (16.2)	56 (16.4)	43 (12.1)	.002
Electrocardiogram (mean ± SD) ms					
Ventricular rate	89.8 ± 26.0	87.5 ± 24.7	92.2 ± 26.9	88.2 ± 25.3	.08
P‐R interval	166.4 ± 36.6	169.7 ± 41.7	166.8 ± 43.0	167.1 ± 40.7	.75
QRS duration	103.9 ± 26.7	107.4 ± 34.9	106.3 ± 29.7	106.3 ± 30.6	.51
QT	400.6 ± 74.9	402.5 ± 70.1	396.3 ± 75.7	398.5 ± 63.4	.70
QTc	474.6 ± 56.1	473.5 ± 52.5	477.5 ± 60.6	467.1 ± 47.8	.08
Laboratory value (mean ± SD)					
Troponin (ng/mL)	11.4 ± 55.2	13.7 ± 45.5	9.48 ± 36.6	3.8 ± 16.6	.023
CKMB (IU/L)	43.9 ± 107.99	60.0 ± 112.5	63.7 ± 149.7	50.4 ± 147.8	.71
Potassium level (mEq/L)	4.3 ± 2.4	4.3 ± 1.6	4.1 ± 0.9	4.1 ± 0.9	.20
Magnesium level (mEq/L)	2.0 ± 0.4	2.0 ± 0.5	2.0 ± 0.4	2.0 ± 0.5	.81
Bicarbonate level (mEq/L)	23.6 ± 5.9	23.6 ± 4.9	23.6 ± 5.1	23.9 ± 5.3	.82
Charlson index‐ (mean ± SD)	2.7 ± 2.4	2.8 ± 2.3	2.6 ± 2.2	2.5 ± 2.3	.42
NYHA class—no. (%)					.76
I	11 (26.2)	22 (48.9)	18 (43.9)	6 (21.4)	
II	13 (31.0)	8 (17.8)	10 (24.2)	12 (42.9)	
III	16 (38.1)	13 (28.9)	13 (31.7)	8 (28.6)	
IV	2 (4.8)	2 (4.4)	0 (0.0)	2 (7.1)	
LV ejection fraction (mean ± SD)	44.7 ± 16.2	43.4 ± 15.9	46.4 ± 15.3	46.6 ± 15.1	.059
SCA rhythm—no. (%)					.72
VT/VF	187 (51.2)	203 (56.5)	180 (52.8)	190 (53.5)	
PEA/asystole	178 (48.8)	156 (43.5)	161 (47.2)	165 (46.5)	
SCA location—no. (%)					.10
In hospital	194 (53.2)	204 (56.8)	192 (56.3)	212 (59.7)	
Out of hospital	171 (46.8)	155 (43.2)	149 (43.7)	143 (40.3)	
Median household income $ (mean ± SD)	34 479.2 ± 6606.9	45 233.2 ± 2667.1	51 974.6 ± 1755.0	73 298.9 ± 14 401.7	<.001

Abbreviations: BMI, body mass index; LV, left ventricle; PEA, pulseless electrical activity; SCA, Sudden cardiac arrest; VT, ventricular tachycardia; VF, ventricular fibrillation.

Of the overall cohort, 47% of patients died during follow‐up. A multivariable logistic regression model was constructed using unbalanced covariates. These included the following variables: age, race, heart rate, QTc, troponin, left ventricular ejection fraction, tobacco use, alcohol use, presence of pulmonary disease, and presence of cerebrovascular disease. In this model, the odds ratio for mortality for those in the lowest income quartile in the logistic regression model was 1.42 (95% confidence interval [CI] 1.02, 1.99) (*P* = 0.041) (Table 2).

**Table 2:** Binary logistic regression examining the independent predictors of long‐term death in survivors of sudden cardiac arrest

	Hazard ratio	95% Confidence interval		*P*‐value
		Lower	Upper	
Lowest quartile by household income	1.42	1.01	1.99	.041
Age (per 1‐year increase)	1.04	1.03	1.05	<.001
Black patients (vs whites)	1.26	0.79	2.01	.34
Heart rate (per 1 beat/min increase)	1.00	1.00	1.01	.06
QTc (per 1 ms increase)	1.00	1.00	1.00	.19
Troponin (per 1 ng/mL)	1.00	0.99	1.00	.06
Left ventricular ejection fraction (per 1% increase)	1.00	0.99	1.00	.76
Tobacco use	0.78	0.57	1.06	.12
Alcohol use	0.83	0.56	1.23	.36
Presence of chronic pulmonary disease	2.09	1.56	2.81	<.001
Presence of cerebrovascular disease	1.53	1.00	2.34	.048

**Figure 1:**
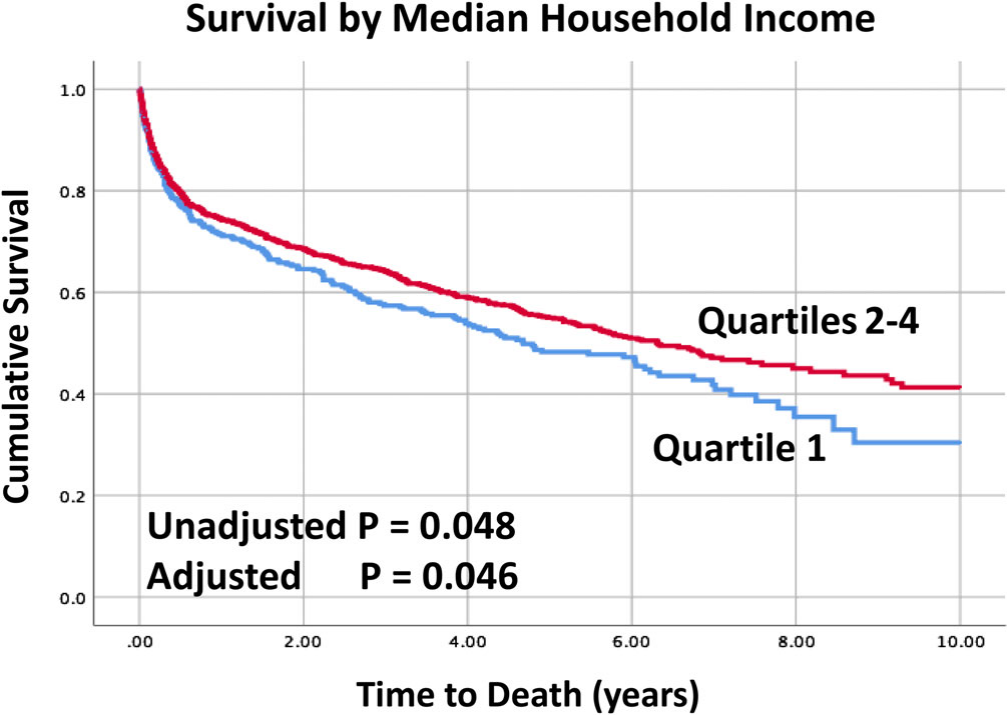
Long‐term survival of victims of sudden cardiac arrest by socioeconomic status

A Cox regression model was generated to determine the hazard ratio for mortality for those in the lowest income quartile. Unbalanced covariates as detailed above were included in the multivariable model. Here again, patients who were in the lowest income quartile were 25% more likely to die with a hazard ratio of 1.25 (95% CI 1.00, 1.56) (*P* = .046) (Table 3, Figure 1).

**Table 3:** Cox proportional hazard model examining the independent predictors of the time to all‐cause mortality in survivors of sudden cardiac arrest

	Hazard ratio	95% Confidence interval		*P*‐value
		Lower	Upper	
Lowest quartile by household income	1.25	1.00	1.56	.046
Age (per 1‐year increase)	1.04	1.03	1.05	<.001
Black patients (vs whites)	1.20	0.88	1.63	.25
Heart rate (per 1 beat/min increase)	1.00	1.00	1.01	.005
QTc (per 1 ms increase)	1.00	1.00	1.00	.72
Troponin (per 1 ng/mL)	1.00	0.99	1.00	.13
Left ventricular ejection fraction (per 1% increase)	1.00	0.99	1.01	.98
Tobacco use	0.94	0.76	1.17	.59
Alcohol use	0.83	0.62	1.11	.21
Presence of chronic pulmonary disease	1.62	1.33	1.95	<.001
Presence of cerebrovascular disease	1.20	0.93	1.53	.17

## 4 DISCUSSION

In this retrospective single‐institution study, we analyzed the impact of SES, as measured by median income of a patient's zip code of residence, on long‐term mortality of SCA survivors. We found that patients in the lowest SES quartile were more likely to die, even when controlling for multiple demographic and clinical risk factors. These findings were separately replicated through both a logistic regression and a Cox regression model. Interestingly, our data demonstrated a threshold effect whereby only patients in the lowest SES quartile had higher mortality. There was no dose‐dependent effect.

Prior work in the field has demonstrated that lower SES is associated with increased incidence of SCA across multiple sites in the United States and Canada.^12^, ^13^ The effect of increased incidence was not completely elucidated but was likely because of the fact that the same factors that cause increased rates of SCA also contribute to increased mortality after SCA.

Although our study was not intended to unmask the mechanisms by which lower SES impacts mortality, there are however several possible links that can be invoked. First, SES in an important social determinant of health that has implications that reach beyond those that can be modified by a physician or hospital system. Patients of lower SES likely have reduced access to healthy foods,^18^, ^19^ less ability to safely exercise,^20^, ^21^ and may have worse mental health.^22^ Prior studies have also demonstrated that patients with lower SES have poor health literacy,^23^ medication adherence,^24^ and higher rates of job stress with associated coronary disease.^25^ Many of these factors can impact patients' outcomes after SCA. Additionally, patients that have suffered from SCA are likely to have a number of important comorbidities, including coronary disease, hypertension, and smoking.^26^

Our study used a purpose‐built dataset of SCA throughout our healthcare system. Our system includes many hospitals ranging from small rural to large urban tertiary centers. Therefore, our data reflect most real‐world hospital practices. Each event was manually curated and adjudicated, allowing for a high degree of reliability. Additionally, generating our dataset allowed us access to detailed clinical and imaging variables not frequently used in retrospective analyses of large datasets.

This study also has limitations. First, it is a retrospective analysis of a single hospital system and therefore may have biases inherent to this type of analysis. To minimize referral bias, we have included all patients presenting to our institution with SCA while minimizing exclusion criteria. Second, we also did not have access to data on frequency of follow‐up, barriers to medical access, and specialty of providers seen at follow‐up, among other variables. Lastly, our indicator of SES was at the level of the zip code area rather than at the individual patient household level. This may have introduced some error in the assigning of patients to the four SES groups. However, prior studies have demonstrated that in the absence of individual‐level data, it is reasonable to use census‐derived data as in our present study. In fact, earlier work suggested that the use of census data may actually underestimate the effect size that would have been observed had individual data been used.^27^ Thus, the association between lower SES and higher mortality may actually be more intimate than what we report.

## 5 CONCLUSION

In this retrospective, single‐system analysis, we demonstrate that the rate of mortality and time to mortality after SCA are higher in patients in the lowest household income quartile. These data support the hypothesis that poor outcomes following SCA are associated with lower SES. Thus, in designing interventions to improve long‐term survival after SCA, measures specifically targeting patients of low SES are necessary and may provide more survival benefit.

## CONFLICT OF INTEREST

The authors declare no potential conflict of interests.
